# Association of objective and subjective parameters of obstructive sleep apnea with plasma aldosterone concentration in 2,066 hypertensive and 25,368 general population

**DOI:** 10.3389/fendo.2022.1016804

**Published:** 2023-01-16

**Authors:** Hui Wang, Mulalibieke Heizhati, Nanfang Li, Lin Gan, Mengyue Lin, Wenbo Yang, Mei Li, Ling Yao, Miaomiao Liu, Adalaiti Maitituersun, Shasha Liu, Zihao Wu, Zuhere Xiamili, Ling Tong, Yue Lin, Qin Luo, Jing Hong

**Affiliations:** Hypertension Center of People’s Hospital of Xinjiang Uygur Autonomous Region, Xinjiang Hypertension Institute, NHC Key Laboratory of Hypertension Clinical Research, Key Laboratory of Xinjiang Uygur Autonomous Region, Hypertension Research Laboratory, Xinjiang Clinical Medical Research Center for Hypertension (Cardio-Cerebrovascular) Diseases, Urumqi, China

**Keywords:** plasma aldosterone concentration, lowest saturation of oxygen, apnea index, no-SAS scale, obstructive sleep apnea, normotension

## Abstract

**Study objectives:**

Obstructive sleep apnea (OSA) severity has been suggested in aldosterone elevation in resistant hypertension, whereas it is undetermined in the rest population. We explored the association of OSA parameters with plasma aldosterone concentration (PAC) in participants with and without hypertension.

**Methods:**

We enrolled clinically hypertensive patients with polysomnography and PAC data under no interfering agents, compared (log) PAC, and assessed the linearity of log PAC by tertiles (T1/2/3) of sleep parameters and their association using linear regression by gender and age. We enrolled participants with and without hypertension who had No-SAS scale and PAC data from the community and duplicated the observations from clinical setting considering age, gender, and presence of hypertension.

**Results:**

Of the 2,066 clinical patients with hypertension (1,546 with OSA), men participants (n=1,412), log apnea–hypopnea index (p=0.043), apnea index (AI, p=0.010), and lowest oxygen saturation (LSaO_2_, p=0.013) showed significant linearity with log PAC. Log AI (B=0.04, 95%CI: 0.01,0.07, p=0.022) and log LSaO_2_ (B=−0.39, 95%CI: −0.78,−0.01, p=0.044) showed significant positive and negative linear associations with log PAC in regression. In community dwellers, 6,417 participants with untreated hypertension (2,642 with OSA) and 18,951 normotensive participants (3,000 with OSA) were included. Of the men participants with and without hypertension, the OSA group showed significantly higher (log) PAC than did their counterparts, and log No-SAS score showed positive association with log PAC (hypertension: B=0.072, 95%CI: 0.002,0.142, p=0.043; normotension: B=0.103, 95%CI: 0.067,0.139, p<0.001) in linear regression analysis, which were consistent in all age groups.

**Conclusions:**

OSA parameters were positively associated with PAC in normotensive and hypertensive participants, indicating that OSA may increase circulating aldosterone, especially in men.

## Introduction

Obstructive sleep apnea (OSA) is a chronic sleep disorder characterized by repetitive episodes of upper airway collapse during sleep, resulting in intermittent hypoxia, hypercapnia, sleep arousal, sleep fragmentation, and daytime sleepiness (1, 2). OSA is independently associated with increased risk for hypertension and cardiovascular morbidity and mortality (3, 4). Approximately 46%–53% of patients with moderate to severe OSA have a diagnosis of hypertension (5). Therefore, OSA represents a tremendous threat to the global health.

OSA is considered as a multi-factorial disease, closely related to genetic, epigenetic environmental, and developmental factors including obesity (6, 7). However, even if the above factors are controlled, OSA still exists (8), suggesting the existence of residual factors affecting the occurrence or severity of OSA. Aldosterone, a steroid hormone produced by adrenal glands and playing important roles in maintaining body fluid and homeostasis, has obtained increasing attention on this aspect recently (9–11).

It has been suggested that aldosterone is involved in OSA and/or its severity through the following suggested mechanisms. Sodium water reabsorption followed by excess aldosterone elevates overnight fluid shifting to the neck in supine position, which results in pharyngeal edema and upper airway obstruction and further contributes to OSA pathogenesis or severity (12). The presence of aldosterone receptors on upper airway smooth muscle cells membranes also supports a direct local role played by aldosterone in increasing para-pharyngeal edema and favoring OSA or aggravating its severity (13). Aldosterone-excess-related potassium and glucose metabolism dysregulation leads to neuropathy development, which affect the central control of respiration and consequently upper airway neural reflexes, further favoring OSA (14). Furthermore, adipose tissue per se and adipocyte-derived factors, such as leptin and complement-C1q TNF-related protein-1, obesity, a well-known risk factor for OSA, induce aldosterone overproduction, independent of renin angiotensin aldosterone system (RAAS) and sympathetic nervous systems (15). Emerging evidence shows that mineralocorticoid receptor antagonists appear to reduce the disease severity in some OSA patients (16, 17). Therefore, it is reasonable to speculate that circulating aldosterone might be involved in OSA severity.

The relationship between OSA and aldosterone in patients with hypertension has been investigated in some studies, reporting a positive correlation between OSA severity and plasma or 24-h urinary aldosterone in resistant hypertension (18–21). However, most studies focused on clinical-resistant hypertension with small sample and failed to exclude effects of interfering agents on aldosterone measurements (9). These limitations might hinder the extrapolation of the results. In addition, the association of OSA severity and aldosterone is less studied in the general population with hypertension (22). Furthermore, studies that assess the association between OSA and plasma aldosterone concentration (PAC) in individuals without hypertension are lacking. In a meta-analysis, researchers performed subgroup analyses separately for participants with and without hypertension (n = 120), and results suggest that OSA is associated with higher PAC in the presence of hypertension (23).

Therefore, to test the hypothesis that there would be linear association between OSA severity parameters and PAC from non-OSA to OSA status in population with and without hypertension, we conducted a gender-stratified cross-sectional study in clinical patients with hypertension who had polysomnography (PSG) data and PAC measurements under no interfering agents and in community dwellers with and without hypertension who had data on No-SAS scale and PAC measured under no interfering agents by considering age, gender, and presence of hypertension. It would be beneficial to identify the association, as the prevalence of OSA remains high and contributes to an increasing risk of adverse outcomes and aldosterone antagonist treatment is effective (16, 17, 24).

## Materials and methods

**Figure 1:**
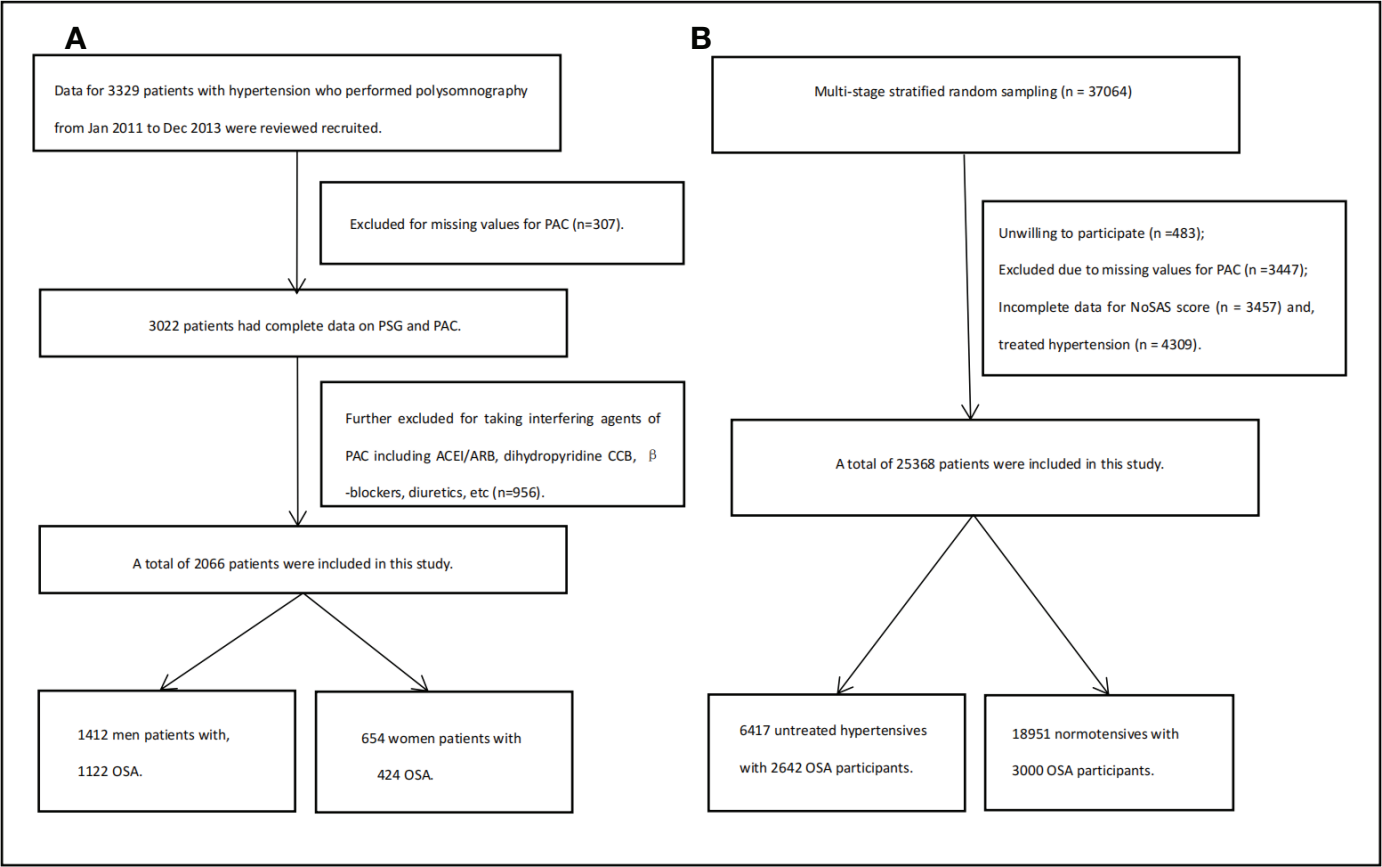
Flow chart for clinical participants **(A)** and for general population **(B)**.

As in the flow chart (**Figure 1A**), in clinical data, participants were the patients in the Urumqi Research on Sleep Apnea and Hypertension (UROSAH) cohort, as described recently (25). Briefly, UROSAH is a single-center, retrospective, observational study to assess the association of OSA with long-term cardiovascular outcomes in patients with hypertension. Data for hypertensive patients aged ≥18 years who visited the Hypertension Center between January 2011 and December 2013 were reviewed. For the current study, we further excluded those with missing PSG and or PAC data and those who underwent PAC measurement when taking antihypertensive agents that would affect RAAS.

As in flow chart (**Figure 1B**), in general population, we used a multi-stage stratified sampling method to enroll study participants aged ≥15 years from Emin county Xinjiang, as described in our previous studies (26–28). For the current study, we excluded participants with hypertension under any antihypertensive agent treatment at the time of survey.

The present study was approved by the Ethics Committee at People’s Hospital of Xinjiang Uygur Autonomous Region. All participants or their legal representatives signed written consent forms.

### Data collection

In clinical data, all patients underwent in-laboratory overnight PSG (Compumedics E series, Australia) examination consistent with our previous studies (29). PSG evaluation included monitoring airflow with nasal pressure, respiratory effort with piezoelectric bands at abdominal and chest locations, oxygen saturation measurement with pulse oximetry, surface electrodes connected with standard techniques to obtain chin electromyography, and electrooculography. Data were scored by sleep technologists licensed by the American Academy of Sleep Medicine. Total sleep time, sleep efficiency, apnea hypopnea index (AHI), apnea index (AI), hypopnea index, mean and lowest oxygen saturation (LSaO_2_), time spent with oxygen saturation <90% (T90), maximum duration of hypoventilation, average duration of hypoventilation, oxygen desaturation index 3 and 4, and wake after sleep onset and arousal index were recorded or calculated. AHI is defined as the total number of hypopneas and apneas that occur per hour of sleep. OSA was defined if AHI was ≥5 events per hour, and mild, moderate, and severe OSA were defined as AHI=5–15, 15–30, and ≥30 events per hour, respectively.

In general population, No-SAS scale was completed in participants by trained investigators. No-SAS score is a recent developed screening tool (30, 31). It ranges from 0 to 17 and allocates 4 points for having a neck circumference of >40 cm, 3 points for having a body mass index (BMI) of 25–30 kg/m^2^, or 5 points for having a BMI of ≥30 kg/m² or higher, 2 points for snoring, 4 points for being >55 years, and 2 points for being male. OSA was defined if the No-SAS score was ≥8 points (30).

### Measurements of PAC in clinical and general population

In clinical data, fasting blood samples were drawn at 9:00 a.m. after patients had been ambulant for at least 2 h and seated for 30 min. Patients were requested to stop taking antihypertensive agents for 4–6 weeks before hormone testing. For patients with BP ≥160/100 mmHg, we switched their antihypertensive agents to alpha-blockers and/or calcium channel blockers (verapamil) for 4–6 weeks before hormone testing. In the presence of severe or symptomatic hypertension, the workup was made under antihypertensive medications known as poorly affecting measurements of PAC and plasma renin activity (PRA). In addition, if patients suffered from hypokalemia, serum potassium (K) was corrected with oral K supplements, as close as possible to 3.9–4.0 mmol/L. All patients were recommended to maintain free diet as usual with no restriction on salt intake.

In the general population, fasting venous blood samples were collected, centrifugated onsite, and transported to Xinjiang Hypertension Institute (located in Urumqi, 500–600 km in distance) in portable refrigerators and were stored at −70°C until measurement in 2021.

PAC was measured using radioimmunoassay (DSL-8600 ACTIVE^®^ Aldosterone Coated Tube Radioimmunoassay Kit; Diagnostic Systems Laboratories, Webster, TX, USA) with the intra- and inter-assay coefficients of variation of 5.6% and 8.5% in both data. In clinical data, PRA was also measured by radioimmunoassay (RIA) using commercial kits (Center of Beifang Biology Technique, Beijing, China), and the intra- and inter-assay coefficients of variation were less than 10% and 15%, respectively. The details of the measurements are described in previous studies from our center (32, 33).

### Other variables

In clinical population, in addition to PSG parameters, PAC, PRA, and aldosterone to renin activity ratio (ARR), data on age, gender, BMI, abdominal circumference, cigarette consumption and alcohol intake (yes/no), diabetes mellitus (DM, yes/no), chronic kidney disease (CKD, yes/no), systolic and diastolic blood pressure (SBP, DBP), fasting plasma glucose (FPG), alanine aminotransferase (ALT), serum creatinine, total cholesterol (TC), triglyceride (TG), serum and 24-h urinary K and sodium, and menopausal status (yes/no) for women participants were included. Seated office BP was measured on the upper arm after patients rested quietly for 10 min at least with a mercury sphygmomanometer using international recommendations at hospitalization. The mean value of two measurements was recorded and used for analysis.

In the general population, data on age, gender, cigarette and alcohol consumption, hypertension, coronary heart disease and stroke, and relevant treatments were also collected. Height, weight, neck and abdominal circumference, and BP were measured. BP was measured three times with a Professional Portable BP Monitor (OMRON HBP-1300, Kyoto, Japan) on the right arm positioned at heart level after the participant was sitting at rest for 5 min, with 30 s between each measurement, and the average of three values was used. Fasting venous blood samples were tested for serum creatinine, FPG, TC, TG, ALT, and aspartate aminotransferase (AST). BMI was calculated as weight (kg)/height^2^ (m).

### Definitions

In both population, hypertension was defined as SBP ≥140 mmHg or DBP ≥90 mmHg, or under antihypertensive therapy. Estimated glomerular filtration rate (eGFR) was calculated using the CKD-EPI equation, and CKD was defined if eGFR<60ml/min/1.73m^2^. DM was defined if fasting glucose ≥7.0 mmol/L, if using any glucose-lowering medication, or if there was self-reported history of diabetes.

In general population data, for men participants, age was categorized into <45, 45–60, and ≥60 years according to the criteria from the World Health Organization for developing countries (25, 27); for women participants, age was categorized into <51 and ≥51 years as a cutoff age for menopausal status based on previous studies (34). Educational attainment status was categorized into <9 and ≥9 years. Alcohol consumption is defined as consuming an alcoholic beverage at least once per week in the past month. Cigarette consumption is defined as smoking at least 20 packets of cigarettes in their lifetime and currently smoking cigarettes. Abdominal obesity is defined as having an abdominal circumference ≥90 cm for men and ≥85 cm for women. Cardiovascular disease is defined as self-reported medical history of coronary heart disease and stroke.

### Statistical analyses

In clinical data, characteristics of participants were provided by non-, mild, moderate, and severe OSA groups. PAC and log PAC were compared by tertiles of PSG-based sleep parameters as T1, T2, and T3 groups by gender (men and women), and linearity of log PAC was explored among the tertile groups. In the general population, participants were divided into two groups by presence of OSA; PAC and log PAC was also compared between non-OSA and OSA groups in age-specific men (<45, 45–60, and ≥60 years) and women participants (<51 and ≥51 years) by untreated hypertension and normotension. Continuous variables with a normal distribution were presented as mean ± standard and compared between groups using analysis of univariable ANOVA test or Student’s t-test; otherwise, they were presented as median (interquartile range, IQR) and compared between groups using nonparametric test. Kolmogorov–Smirnov test was used to check the data normality. We provided both PAC and log PAC for among- or between-group comparison. Categorical variables were expressed as frequency and percentage, and chi-square test was used for among- or between-group comparison. Multivariate linear regression was used to evaluate the relationship of log-transformed PSG sleep parameters and log No-SAS score with log PAC by calculating B and 95% confidence intervals (95%CI) due to their skewed distribution. Variables to be adjusted were selected using univariate linear regression (p<0.10). Tolerance and variance inflation factor of multiple linear regression were used to evaluate collinearity among variables. Collinearity was considered when tolerance <0.1 or variance inflation factor >10. Data were analyzed using SPSS statistical software (version 25.0, SPSS Inc., Chicago, IL). All analyses were two-tailed, and p-value<0.05 was statistically significant.

## Results

### Clinical population

As given in the flow chart (**Figure 1A**), of the 3,329 participants in UROSAH, 2,066 participants with hypertension, who underwent PAC measurement under no interfering agents, comprising the current analytical sample aged 46 years and 68.3% (n=1,412) were men. There were 520 non-OSA, 627 mild, 465 moderate, and 454 severe OSA participants (**Table 1**).

**Table 1:** Characteristics of hypertensive participants by total and by severity of obstructive sleep apnea (OSA) with measured plasma aldosterone under no interfering agents in UROSAH cohort.

	Total	Non-OSA	Mild OSA	Moderate OSA	Severe OSA	p
N	2,066	520 (25.2)	627 (30.3)	465 (22.5)	454 (22.0)	
Age (years)	46.0 (40.0,52.0)	43.0 (37.0,48.0)	47.0 (41.0,52.0)	48.0 (41.0,54.0)	47.0 (40.0,53.0)	<0.001
Men (n,%)	1412.0 (68.3)	290.0 (55.8)	394.0 (62.8)	346.0 (74.4)	382.0 (84.1)	<0.001
Women (n,%)	654 (31.7)	230 (44.2)	233 (37.2)	119 (25.6)	72 (15.9)	<0.001
Cigarette consumption (n,%)	735.0 (35.6)	133.0 (25.6)	213.0 (34.0)	181.0 (38.9)	208.0 (45.8)	<0.001
Alcohol intake (n,%)	730.0 (35.3)	146.0 (28.1)	208.0 (33.2)	171.0 (36.8)	205.0 (45.2)	<0.001
Diabetes (n,%)	271.0 (13.1)	40.0 (7.7)	82.0 (13.1)	67.0 (14.4)	82.0 (18.1)	<0.001
Chronic kidney disease (n,%)	70.0 (3.4)	13.0 (2.5)	22.0 (3.5)	23.0 (4.9)	12.0 (2.6)	0.139
Body mass index (kg/m^2^)	27.6 (25.4,30.1)	26.2 (24.1,28.6)	27.3 (25.4,29.8)	28.1 (25.9,30.7)	29.0 (26.7,31.5)	<0.001
Abdominal circumference (cm)	99.0 (92.0,105.0)	94.0 (87.0,100.0)	98.0 (92.0,104.0)	101.0 (95.0,107.0)	103.0 (97.0,110.0)	<0.001
ALT (U/L)	24.0 (17.0,35.0)	23.0 (16.0,33.0)	24.0 (17.0,35.0)	22.0 (16.0,33.0)	26.0 (18.0,36.0)	<0.001
eGFR (ml/min/1.73 m^2^)	100.4 (86.2,109.9)	103.5 (87.6,113.0)	99.3 (85.4,108.8)	98.7 (87.6,107.9)	101.2 (86.5,110.3)	0.002
Fasting plasma glucose (mmol/L)	4.9 (4.5,5.3)	4.8 (4.4,5.3)	4.9 (4.5,5.3)	4.9 (4.5,5.3)	5.0 (4.5,5.5)	0.018
Serum potassium (mmol/L)	3.9 (3.7,4.1)	4.0 (3.7,4.1)	3.9 (3.7,4.1)	3.9 (3.7,4.1)	3.9 (3.7,4.1)	0.006
Serum sodium (mmol/L)	141.0 (139.0,143.0)	141.0 (139.0,143.0)	141.0 (139.0,143.0)	141.0 (139.0,143.0)	141.0 (139.0,143.0)	0.417
Plasma renin activity (ng/ml per h)	1.4 (0.6,2.7)	1.5 (0.7,2.8)	1.3 (0.6,2.6)	1.3 (0.6,2.7)	1.5 (0.6,2.7)	0.388
Plasma aldosterone concentration (ng/dl)	13.5 (9.2,19.6)	13.1 (9.0,18.8)	13.2 (9.0,19.7)	13.8 (9.1,20.3)	14.2 (9.7,19.5)	0.206
Aldosterone renin ratio	9.8 (5.0,21.4)	9.1 (4.6,19.1)	9.9 (5.1,21.4)	10.7 (52,23.4)	9.5 (5.3,22.9)	0.223
Total cholesterol (mmol/L)	4.5 (3.9,5.0)	4.4 (3.8,5.0)	4.5 (3.8,5.1)	4.8 (3.9,5.0)	4.5 (4.0,5.1)	0.515
Triglyceride (mmol/L)	1.7 (1.2,2.4)	1.6 (1.1,2.2)	1.7 (1.2,2.4)	1.8 (1.2,2.4)	1.9 (1.3,2.6)	<0.001
Systolic blood pressure (mmHg)	140.0 (130.0,150.0)	140.0 (126.0,150.0)	140.0 (130.0,150.0)	140.0 (130.0,150.0)	140.0 (130.0,150.0)	0.046
Diastolic blood pressure (mmHg)	90.0 (83.4,100.0)	90.0 (82.0,100.0)	90.0 (80.0,100.0)	90.0 (82.0,100.0)	94.0 (88.0,100.0)	0.025
Total sleep time (min)	379.0 (334.0,419.0)	379.8 (333.6,422.0)	375.0 (331.0,413.0)	371.5 (329.5,408.3)	389.5 (341.4,429.5)	0.001
Sleep efficiency (%)	72.0 (63.2,79.9)	71.5 (63.0,80.0)	71.6 (63.1,78.9)	70.1 (61.8,78.8)	74.8 (65.6,81.9)	<0.001
24 h urinary potassium (mmol/l)	34.0 (23.7,44.2)	32.9 (23.7,42.8)	34.0 (24.4,44.1)	35.5 (22.3,45.3)	34.5 (23.7,45.4)	0.362
24 h urinary sodium (mmol/l)	164.8 (106.1,230.9)	159.0 (106.2,220.6)	166.1 (106.4,232.2)	165.3 (103.8,239.2)	171.2 (105.1,235.8)	0.537
Apnea hypopnea index (event/h)	12.3 (4.8,27.0)	1.6 (0.7,3.0)	8.9 (6.6,11.6)	20.4 (17.6,25.0)	46.5 (6.0,60.8)	<0.001
Apnea index (event/h)	1.1 (0.1,7.1)	0.0 (0.0,0.2)	0.6 (0.1,2.0)	3.6 (0.9,8.2)	21.2 (8.5,36.4)	<0.001
Hypopnea index (event/h)	8.5 (3.4,17.0)	1.4 (0.5,2.7)	7.3 (5.5,9.9)	16.2 (12.2,19.6)	24.8 (14.0,34.4)	<0.001
Lowest desaturation of oxygen (%)	82.0 (77.0,87.0)	88.0 (87.0,90.0)	83.0 (80.0,86.0)	80.0 (75.0,83.0)	74.0 (67.0,78.0)	<0.001
Mean desaturation of oxygenSaO2 (%)	93.0 (91.0,94.0)	94.0 (93.0,95.0)	93.0 (92.0,94.0)	92.0 (91.0,94.0)	91.0 (90.0,93.0)	<0.001
T90 (min)	9.4 (1.0,50.3)	0.4 (0.0,1.6)	6.7 (2.0,21.1)	22.0 (8.4,52.9)	73.9 (30.9,129.2)	<0.001
Oxygen desaturation index ≥3% (event/h)	28.7 (15.4,49.2)	11.9 (7.3,18.4)	24.0 (18.2,30.9)	38.9 (31.9,48.5)	67.2 (53.4,84.5)	<0.001
Oxygen desaturation index ≥4% (event/h)	15.8 (6.7,32.1)	4.6 (2.5,7.4)	12.7 (9.0,16.8)	25.0 (19.9,30.9)	52.2 (40.0,70.4)	<0.001
Maximum hypoventilation duration (min)	33.9 (25.4,44.2)	22.1 (17.6,27.9)	31.6 (26.4,38.9)	39.0 (32.6,47.3)	45.8 (37.4,56.4)	<0.001
Average hypoventilation duration (min)	19.6 (17.5,22.1)	17.4 (15.3,19.7)	19.5 (17.9,21.8)	20.5 (18.5,22.9)	21.2 (18.6,24.1)	<0.001
Wake after sleep onset (min)	62.5 (33.5,101.5)	61.0 (34.0,98.4)	63.8 (33.9,104.1)	67.0 (37.3,105.8)	55.3 (,30.5,94.8)	0.015
Arousal index (min)	14.0 (9.0,20.0)	13.0 (9.0,18.0)	13.5 (9.0,9.0,19.0)	14.0 (9.0,20.0)	14.0 (8.3,21.0)	0.594

OSA, obstructive sleep apnea; UROSAH, Urumqi Research on Sleep Apnea and Hypertension; ALT, alanine aminotransferase; eGFR, estimated glomerular filtration rate; T90, time spent with oxygen saturation <90%.

As in **Table 2**, in men participants, the T3 group of AI (the highest tertile T3 *vs*. the lowest T1: 14.5 *vs*. 13.0 ng/dl, p=0.027) and of LSaO_2_ (the lowest T3 *vs*. the highest T1: 14.1 *vs*. 12.9 ng/dl, p=0.016) showed significantly higher PAC, compared with the T1 group, and log PAC showed significant linearity among tertiles of AHI (p=0.043), AI (p=0.010), and LSaO_2_ (p=0.013), whereas these were not in women participants (**Supplementary Table S1**). Sample size and range of PAC by tertiles of PSG parameters are provided in **Supplementary Table S2**.

**Table 2:** Comparison of plasma aldosterone concentration by tertile of polysomnography sleep parameters in men participants with hypertension under no interfering agents with Bonferroni correction for between group comparison.

Parameters	T1	T2	T3	p	p1	p2	p3	p for trend
Log PAC (mean ± SD)								
AHI tertile	1.11 ± 0.22	1.13 ± 0.23	1.14 ± 0.23	0.125	0.682	0.128	1.00	0043
AI tertile	1.11 ± 0.22	1.12 ± 0.23	1.15 ± 0.22	0.035	0.875	0.030	0.383	0.010
HI tertile	1.12 ± 0.22	1.13 ± 0.23	1.12 ± 0.23	0.425	0.581	1.00	1.00	0.601
LSaO2 tertile	1.10 ± 0.23	1.13 ± 0.23	1.14 ± 0.22	0.044	0.519	0.039	0.849	0.013
MSaO2 tertile	1.12 ± 0.22	1.13 ± 0.22	1.13 ± 0.23	0.670	1.000	1.000	1.000	0.384
T90% tertile	1.13 ± 0.22	1.12 ± 0.23	1.13 ± 0.23	0.509	1.000	1.000	1.000	0.831
ODI3 tertile	1.13 ± 0.22	1.12 ± 0.23	1.13 ± 0.23	0.658	0.754	0.622	0.970	0.819
ODI4 tertile	1.13 ± 0.22	1.11 ± 0.24	1.13 ± 0.22	0.401	0.947	0.642	0.481	0.825
Max hypo duration tertile	1.12 ± 0.23	1.12 ± 0.23	1.14 ± 0.22	0.162	1.000	0.361	0.251	0.119
Aver hypo duration tertile	1.12 ± 0.23	1.13 ± 0.23	1.13 ± 0.22	0.501	0.818	1.000	1.000	0.359
WASO tertile	1.12 ± 0.21	1.13 ± 0.22	1.12 ± 0.24	0.447	0.662	1.000	1.000	0.746
Arousal index	1.12 ± 0.22	1.13 ± 0.23	1.13 ± 0.22	0.630	1.000	1.000	1.000	0.640
PAC (median, IQR)								
AHI tertile	13.1 (9.0,18.9)	13.3 (9.2,19.8)	14.2 (9.6,19.6)	0.435	0.512	0.555	0.198	/
AI tertile	13.0 (8.9,18.9)	13.0 (8.8,19.1)	14.5 (10.1,19.7)	0.032	0.270	0.027	0.053	/
HI tertile	13.1 (9.1,18.5)	13.9 (9.4,20.1)	13.6 (9.1,19.6)	0.250	0.114	0.972	0.126	/
LSaO_2_ tertile	12.9 (8.7,19.0)	13.7 (9.5,19.7)	14.1 (10.1,19.6)	0.046	0.806	0.016	0.068	/
MSaO_2_ tertile	13.2 (9.3,18.8)	13.4 (9.1,19.6)	14.3 (9.1,20.0)	0.550	0.851	0.465	0.252	/
T90% tertile	13.3 (9.1,19.1)	13.2 (9.2,19.7)	13.9 (9.1,19.5)	0.731	0.628	0.548	0.628	/
ODI ≥3% tertile	13.3 (9.1,19.8)	13.3 (9.1,19.0)	13.8 (9.3,19.6)	0.764	0.832	0.795	0.952	/
ODI ≥4% tertile	13.5 (9.1,19.7)	13.1 (9.0,19.2)	13.9 (9.5,19.5)	0.548	0.786	0.445	0.589	/
Max hypo duration tertile	13.5 (8.9,19.6)	13.3 (9.0,19.7)	13.7 (9.9,19.5)	0.198	0.856	0.140	0.098	/
Aver hypo duration tertile	13.5 (8.7,19.5)	13.4 (9.1,19.6)	13.6 (9.9,19.5)	0.054	0.315	0.246	0.011	/
WASO	13.4 (9.2,19.0)	13.9 (9.5,20.1)	13.2 (8.9,19.2)	0.726	0.662	0.428	0.758	/
Arousal index	13.5 (9.2,19.0)	13.6 (9.5,19.8)	13.3 (8.9,19.5)	0.528	0.625	0.465	0.338	/

PAC, plasma aldosterone concentration; AHI, apnea hypopnea index; AI, apnea index; HI, hypopnea index; LSaO_2_, lowest saturation of oxygen; MSaO_2_, mean saturation of oxygen; T90, time spent with oxygen saturation <90%; ODI, Oxygen Desaturation Index; hypo, hypoventilation; WASO, wake after sleep onset p, for among group comparison; p1, for T1 vs. T2 comparison; P2, for T1 vs. T3 comparison; P3, for T2 vs. T3 comparison.

As in **Table 3**, in the linear regression analysis for men participants, log AI showed significant positive association with log PAC in the crude model (B=0.02, 95%CI: 0.01,0.04, p=0.049), and the association became stronger in the model (B=0.04, 95%CI: 0.01,0.07, p=0.022) adjusted for log age, BMI, SBP, DBP, ALT, PRA, and serum and 24-h urinary K, which were selected using univariate linear regression and tested for collinearity (**Supplementary Table S3**). In addition, log LSaO_2_ showed negative association with log PAC marginally in crude model (B=−0.17, 95%CI: −0.35,0.01, p=0.067) and significantly in the adjusted model for the above variables (B=−0.39, 95%CI: −0.78,−0.01, p=0.044). Moreover, log T90 showed marginally positive association with log PAC in adjusted model (B=0.02, 95%CI: −0.01,0.05, p=0.083).

**Table 3:** Liner regression analysis for the relationship of log-transformed PSG-based sleep parameters with log-transformed plasma aldosterone concentration in men participants with hypertension without interfering agents (B, 95%CI, p).

Log plasma aldosterone concentration	Crude model	Adjusted model
Log apnea hypopnea index	0.01 (−0.01,0.04), 0.146	0.01 (−0.03,0.05), 0.738
Log apnea index	0.02 (0.01,0.04), 0.049	0.04 (0.01,0.07), 0.022
Log hypopnea index	0.01 (−0.02,0.03), 0.757	−0.04 (−0.08,0.01), 0.129
Log lowest desaturation of oxygen	−0.17 (−0.35,0.01), 0.067	−0.39 (−0.78,−0.01), 0.044
Log mean oxygen saturation	−0.12 (−0.60,0.36), 0.638	0.12 (−0.48,0.86), 0.582
Log time spent with oxygen saturation <90%	0.01 (−0.01,0.02), 0.540	0.02 (−0.01,0.05), 0.083
Log oxygen desaturation index ≥3%	0.00 (−0.03,0.03), 0.994	−0.01 (−0.05,0.02), 0.454
Log oxygen desaturation index ≥4%	0.01 (−0.02,0.03), 0.739	−0.01 (−0.03,0.02), 0.642
Log maximum duration of hypoventilation	0.03 (−0.02,0.09), 0.245	0.04 (−0.07,0.15), 0.509
Log average duration of hypoventilation	0.03 (−0.05,0.12), 0.472	0.04 (−0.13,0.21), 0.653
Log wake after sleep onset	−0.01 (−0.04,0.02), 0.642	0.02 (−0.03,0.08), 0.410
Log arousal time	0.01 (−0.03,0.056), 0.707	0.02 (−0.05,0.10), 0.575

Adjusted for log-transformed age, body mass index, systolic and diastolic blood pressure, alanine aminotransferase, plasma renin activity, serum potassium, and 24-h urinary potassium

### Community-based population

As given in the flow chart (**Figure 1B**), 6,417 participants with untreated hypertension (2,642 with OSA) and 18,951 participants without hypertension (3,000 with OSA) with complete No-SAS scale and PAC data were included (**Table 4**).

**Table 4:** Characteristics of non-OSA and OSA participants by presence of hypertension in community dwellers.

	Untreated hypertension			Normotension		
	Non-OSA	OSA	p	Non-OSA	OSA	p
N	3,775	2,642		15,951	3,000	
Gender, men, n,%	1,771 (46.9)	2,059 (77.9)	<0.001	5,379 (33.7)	2,449 (81.6)	<0.001
Women,n,%	2,004 (53.1)	583 (22.1)		10,572 (66.3)	551 (18.4)	
Age (years)	49.0 (25.0,75.0)	58.0 (47.0,66.0)	<0.001	41.0 (32.0,49.0)	56.0 (40.0,56.0)	<0.001
<45 years (n,%)	1,177 (31.2)	538 (20.4)	<0.001	9,469 (59.4)	953 (31.8)	<0.001
45–60 years (n,%)	1,910 (50.6)	935 (35.4)		5,576 (35.0)	1,068 (35.6)	
≥60 years (n,%)	688 (18.2)	1,169 (44.2)		906 (5.7)	979 (32.6)	
Education <9 years (n,%)	2,926 (78.2)	1,906 (74.8)	0.003	10,400 (66.2)	1,965 (67.3)	0.039
≥9 years	814 (21.8)	640 (25.1)		5,320 (33.8)	954 (32.7)	
Cigarette use (yes, n,%)	999 (26.6)	925 (36.4)	<0.001	3,263 (20.5)	1,361 (46.8)	<0.001
Alcohol intake (yes, n,%)	1,267 (33.8)	1,269 (50.1)	<0.001	4,002 (25.2)	1,517 (52.2)	<0.001
CVD (n,%)	90 (2.4)	107 (4.0)	<0.001	194 (1.2)	97 (3.2)	<0.001
Neck circumference (cm)	35.4 (33.4,37.5)	40.1 (37.4,42.0)	<0.001	34.2 (32.2,36.5)	40.0 (37.6,42.1)	<0.001
Body mass index (kg/m2)	24.9 (23.0,27.5)	29.1 (26.7,31.8)	<0.001	23.8 (21.6,26.2)	28.2 (26.1,30.9)	<0.001
AC (cm)	88.3 (82.0,94.6)	100.0 (93.6,106.5)	<0.001	83.4 (76.8,90.4)	97.8 (92.0,104.0)	<0.001
Abdominal obesity (n,%)	2,050 (54.4)	2,266 (87.7)	<0.001	5,869 (37.1)	2,485 (83.8)	<0.001
Systolic BP (mmHg)	143.0 (134.0,152.0)	146.0 (138.0,158.0)	<0.001	115.0 (107.0,123.0)	122.0 (115.0,130.0)	<0.001
Diastolic BP (mmHg)	91.0 (85.0,96.0)	92.0 (86.0,98.0)	<0.001	74.0 (69.0,80.0)	78.0 (70.0,82.0)	<0.001
FPG (mmol/L)	5.31 (4.84,5.89)	5.53 (5.00,6.30)	<0.001	5.16 (4.71,5.64)	5.41 (4.90,6.06)	<0.001
ALT (U/L)	22.0 (16.0,29.5)	23.0 (17.0,31.0)	<0.001	19.0 (15.0,26.0)	22.0 (17.0,30.0)	<0.001
AST (U/L)	22.0 (19.0,27.0)	22.0 (18.0,28.0)	<0.001	21.0 (17.0,25.0)	22.0 (18.0,26.0)	0.896
TC (mmol/L)	4.90 (4.20,5.70)	5.00 (4.30,5.70)	<0.001	4.50 (3.88,5.30)	4.80 (4.10,5.60)	<0.001
TG (mmol/L)	1.24 (0.90,1.80)	1.48 (1.00,2.17)	<0.001	1.10 (0.80,1.60)	1.40 (1.00,2.00)	<0.001
Serum Cr (mmol/l)	69.0 (58.7,79.9)	72.7 (61.2,84.9)	<0.001	67.6 (56.8,79.0)	72.0 (61.9,84.0)	<0.001
No-SAS score	4.0 (2.0,6.0)	11.0 (9.0,11.0)	<0.001	2.0 (0.0,5.0)	9.0 (9.0,11.0)	<0.001

OSA, obstructive sleep apnea; CVD, cardiovascular disease; AC, abdominal circumference; BP, blood pressure; FPG, fasting plasma glucose; TC, total cholesterol; TG, triglyceride; Cr, creatinine; ALT, alanine aminotransferase; AST, aspartate aminotransferase.

As in **Table 5**, in the OSA group, young and middle-aged men participants showed significantly higher PAC and log PAC in untreated hypertension and in nornotension. However, women participants (1,603 post-menopausal status) with OSA and untreated hypertension showed significantly lower PAC and log PAC, compared with their counterparts.

**Table 5:** Comparison of PAC and log PAC between total, men, and women participants with and without OSA by presence of hypertension and by age in community-based data.

	Untreated hypertension			Normotension		
	Non-OSA	OSA	P	Non-OSA	OSA	P
N	3,775	2,642		15,951	3,000	
PAC (median, interquartile range)						
Men	12.2 (8.7,17.5)	13.4 (9.3,19.2)	<0.001	13.0 (9.2,18.4)	14.4 (10.1,20.4)	<0.001
<45 years	12.8 (9.1,18.4)	14.0 (9.5,20.0)	0.025	12.9 (9.1,18.4)	15.6 (10.6,21.0)	<0.001
45–60 years	12.2 (8.6,17.4)	14.0 (9.6,20.0)	<0.001	13.2 (9.3,18.5)	14.9 (10.3,21.1)	<0.001
≥60 years	10.3 (7.9,15.8)	12.5 (8.9,17.3)	<0.001	12.7 (9.1,17.5)	13.0 (9.4,18.2)	0.403
Women	15.0 (10.8,20.9)	14.2 (10.5,19.7)	0.010	16.0 (11.2,23.1)	14.0 (10.6,18.9)	<0.001
<51 years	17.3 (12.2,22.8)	16.3 (11.4,23.6)	0.007	16.6 (11.5,24.1)	17.8 (11.7,23.4)	0.841
≥51 years	14.6 (10.7,20.3)	14.10 (10.5,19.3)	<0.001	14.3 (10.7,19.9)	14.0 (10.4,18.5)	0.061
Log plasma aldosterone concentration (mean ± standard deviation)						
Men	2.09 ± 0.20	2.13 ± 0.20	<0.001	2.12 ± 0.20	2.16 ± 0.20	<0.001
<45 years	2.11 ± 0.20	2.14 ± 0.20	0.043	2.12 ± 0.20	2.18 ± 0.20	<0.001
45-60 years	2.09 ± 0.20	2.15 ± 0.20	<0.001	2.12 ± 0.20	2.17 ± 0.20	<0.001
≥60 years	2.05 ± 0.20	2.10 ± 0.19	0.001	2.11 ± 0.20	2.13 ± 0.20	0.362
Women	2.18 ± 0.20	2.17 ± 0.20	0.015	2.22 ± 0.23	2.16 ± 0.19	<0.001
<51 years	2.23 ± 0.23	2.20 ± 0.20	0.001	2.23 ± 0.23	2.23 ± 0.18	0.915
≥51 years	2.12 ± 0.18	2.17 ± 0.19	<0.001	2.16 ± 0.19	2.15 ± 0.19	0.043

PAC, plasma aldosterone concentration.

In the linear regression analysis shown in **Table 6**, log No-SAS score showed significant positive association with log PAC in men with hypertension (B=0.068, 95%CI: 0.044,0.092, p<0.001) and without hypertension (B=0.060, 95%CI: 0.045,0.076, p<0.001) in the crude model and in the adjusted model (hypertension: B=0.072, 95%CI: 0.002,0.142, p=0.043; normotension: B=0.103, 95%CI: 0.067,0.139, p<0.001) for variables age, education attainment status, cigarette consumption, alcohol intake, cardiovascular disease, SBP, DBP, BMI, abdominal circumference, FPG, serum creatinine, TC, TG, ALT, and AST selected using univariate regression analysis (**Supplementary Table S4**).

**Table 6:** Liner regression for the relationship of log No-SAS score with and log plasma aldosterone concentration in total, men, and women participants by presence of hypertension and by age in community-based participants (B, 95%CI, p).

	Crude model	Adjusted model
Untreated hypertension		
Men	0.068 (0.044,0.092), <0.001	0.072 (0.002,0.142), 0.043
<45 years	0.058 (0.020,0.095), 0.003	0.093 (0.023,0.162), 0.009
45–60 years	0.105 (0.069,0.141), <0.001	0.155 (0.096,0.214), <0.001
≥60 years	0.212 (0.129,0.294), <0.001	0.295 (0.162,0.429), <0.001
Women	−0.035 (−0.063,−0.006), 0.017	0.030 (−0.029,0.090), 0.316
<51 years	0.039 (−0.026,0.104), 0.242	0.145 (0.015,0.275), 0.028
≥51 years	−0.052 (−0.101,−0.003), 0.038	0.011 (−0.062,0.083), 0.773
Normotension		
Men	0.060 (0.045,0.076), <0.001	0.103 (0.067,0.139), <0.001
<45 years	0.066 (0.044,0.087), <0.001	0.100 (0.063,0.137), <0.001
45–60 years	0.086 (0.059,0.112), <0.001	0.087 (0.045,0.129), <0.001
≥60 years	0.111 (0.010,0.211), 0.031	0.267 (0.117,0.416), 0.020
Women	−0.106 (−0.134,−0.078), <0.001	0.009 (−0.029,0.047), 0.654
<51 years	−0.071 (−0.116,−0.027), 0.002	0.048 (−0.020,0.116), 0.165
≥51 years	−0.053 (−0.090,−0.016), 0.005	0.033 (−0.054,0.054), 0.998

Adjusted for log-transformed age, systolic and diastolic blood pressure, body mass index, abdominal circumference, fasting blood glucose, serum creatinine, total cholesterol, triglyceride, alanine aminotransferase, aspartate aminotransferase, and education, cigarette consumption, alcohol intake, and cardiovascular disease

This association between log No-SAS score and log PAC in men participants with and without hypertension also stayed consistent in young, middle-aged, and old age groups.

In women participants, log No-SAS score showed significant association with log PAC only in the aged <51 years with hypertension after adjustment for above variables (B=0.145, 95%CI: 0.015,0.275, p=0.028).

## Discussion

Elevated circulating aldosterone has consistently been shown to be a risk factor for vascular disease (10, 33, 35–37) and renal dysfunction (32) in various population. It is undetermined whether circulating aldosterone is elevated in OSA, a well-known risk factor for CVD (3, 4). Therefore, we assessed the association of objective and subjective OSA parameters with PAC in hypertensive and normotensive general population in this cross-sectional study.

We observed the following. First, men with hypertension and with the highest and the lowest tertile of AI and LSaO_2_ contain higher PAC, and log PAC showed significant linearity with AHI, AI, and LSaO_2_. Second, log AI and log LSaO_2_ showed significant positive and negative association with log PAC in crude and adjusted linear regression analysis; log T90 showed marginally positive association with log PAC in adjusted model. Third, in community dwellers, the OSA group in young and middle-aged men participants showed significantly higher PAC and log PAC in stratification by untreated hypertension and by nornotension. Fourth, in linear regression analysis, log No-SAS score showed significant positive association with log PAC in men participants with and without hypertension, which is consistent in young, middle-aged, and old age groups. Fifth, in women participants from the community, log No-SAS score showed significant association with log PAC only in those aged <51 years with hypertension after adjustment for confounding variables.

Current observations may add some evidence on uncertainty on the association of OSA with circulating aldosterone and extend previous observations in resistant hypertension OSA to general hypertensive and normotensive population, in which PAC was measured under no interfering antihypertensive agents. That is, consistent with previous studies mainly conducted in resistant hypertension (18–20), PSG-based objective (AI, LSaO_2_) and questionnaire-based subjective (No-SAS score) OSA parameters showed significant linear association with PAC in participants with and without hypertension from the clinical setting and from the community.

In clinical data, one of the differences in the present study is that PAC was measured under no interfering agents in patients included in the study, which previous studies failed to consider (18–20). Based on current evidence, most antihypertensive agents affect PAC measurement (29) or even increase the PAC (38). The other difference is that we performed this study in general hypertension clinical and community setting, since those from the clinical setting were given antihypertensive agent, which would not affect RAAS at the time of PAC measurement, indicating that most of them were not resistant hypertension. In addition, all the participants with hypertension included from the community were untreated at the time of blood sample collection. Total and all age group men with hypertension and OSA showed significantly higher PAC and log PAC than did those without OSA. In addition, a positive independent linear association between log No-SAS score and log PAC was observed, consistent in age-stratified analysis. Previous relevant studies were conducted mainly in resistant hypertension, in which PA accounts for as high as 36% (25) and as high as 22.0% of general hypertension (26, 27). These results are consistent with those of our clinical data and with those of previous studies (18–20, 24). Therefore, these factors may make the current results more convincing.

In normotensive population from the community, more importantly, total and all age group men with OSA showed significantly higher PAC than did those without OSA, and regression analysis showed a positive independent linear association of log No-SAS score with log PAC in total men participants. The results are consistent with those of a study conducted in resistant hypertensive patients, which showed that those at high risk for OSA by Berlin Questionnaire are characterized by increased 24-h urinary aldosterone excretion (21). This observation might be of significance when considering the harmful effects of both aldosterone and OSA. Previous studies have reported that higher circulating aldosterone leads to elevation in BP and to the development of hypertension (39); current results may implicate that elevated aldosterone in normotensive OSA population might exert some roles in BP elevation and hypertension development in this specific population group. In addition, OSA population without hypertension also showed increased risk for CVD, and thus, it might be reasonable to speculate that elevated circulating aldosterone in this specific population may also play some role in this process, based on previous studies that elevation in circulating aldosterone is a risk factor for CVD (10, 35–37). However, longitudinal studies are needed, especially in those without hypertension or other CV risk factors at baseline, since most relevant studies are performed in hypertensive or high-risk population (10, 35–37). Several studies reported that mineralocorticoid antagonists could improve BP control and OSA severity (16, 28–30). Therefore, aldosterone blockade may be a useful strategy as a supplementary treatment for hypertensive patients with OSA, but further research is still needed to prove it.

We considered the effects of gender on the results. In women participants in total, pre- and post-menopausal status from clinical data, we failed to generate an association between sleep parameters and PAC. However, in our clinical sample, men participants account for 66.1%, possibly making the women sample under-powered to observe a thin kind subtle association. In population-based data, a positive linear association was observed between log No-SAS score with log PAC in the participants aged <51 years with hypertension after adjustment for confounders. We selected 51 years as the cutoff for age stratification analysis in women participants, since we considered that this age might be a marker for pre- and post-menopausal status, based on previous studies (34). Therefore, this association may indicate that female patients with hypertension and OSA may be able to affect aldosterone secretion. In addition, previous studies show that aldosterone secretion in older population is prone to be independent of regulators such as adrenocorticotropic hormone, renin, and K due to aldosterone-producing cluster cells in the adrenal gland (40). Therefore, in older women participants with hypertension and OSA, the effects of aldosterone-producing cell clusters on PAC may outweigh that of OSA. However, further studies are needed.

In the current study, we observed an independent association between LSaO_2_ (significantly) and T90 (marginally after adjustment for confounding variables) with circulating aldosterone, consistent with previous studies. Previous human, animal, and *in vitro* studies show that chronic intermittent hypoxia, a pathophysiological manifestation in OSA, induces sympathetic outflow to the kidney, stimulates renin release, and leads to elevated circulating levels of angiotensin II (Ang II) (41, 42), both of which are important regulators for aldosterone secretion from the adrenal gland. In addition, it has been shown that Ang II is higher in OSA patients than in healthy controls (23). In addition, we observed that AI is in significant positive linear association with PAC, partially consistent with previous studies (18–20). Indeed, AI is a part of AHI, a mechanistic complete obstruction of upper airway during sleep in OSA, followed by hypoxic events and lowering of circulating oxygen saturation, which might be the trigger for increase in circulating aldosterone. In addition, there might be a bidirectional relationship between OSA and circulating aldosterone. OSA stimulates aldosterone secretion, which in turn leads to excess fluid retention and displacement of fluid from the lower extremities to the neck during sleep, resulting in upper airway narrowing and or obstruction (43). In another study conducted in patients with diastolic heart failure, hypertension, and OSA, diuretic treatment improves upper airway caliber and OSA severity, further implicating pharyngeal edema as a cause of upper airway collapse during sleep (44). Furthermore, aldosterone, related to sarcopenia (45), might affect the mass and function of upper dilator muscles, and lower skeletal muscle mass was found to be higher in patients with primary aldosteronism (46). Therefore, elevated aldosterone may also increase the apnea events through above mechanisms. Luckily, in a recent study of mice model, the non-steroidal mineralocorticoid receptor antagonist (finerenone) brought significant improvements in clinically relevant functional parameters in skeletal muscle (47).

The following aspects may merit the current study. First, we enrolled patients with hypertension with a larger sample from clinical and community settings, in which PAC was measured under no interfering agents, and we considered the effects of gender and age, which may reflect objectiveness of current results. Second, we replicated the clinical observation in large community-based population and extended previous observations to normotensive population. Third, we considered important regulators of PAC including PRA and 24-h UK, reflecting K intake, and confounders like 24-h urinary sodium. However, the current study contains some limitations, which should be considered when interpreting the observations. First, the cross-sectional nature of the study would not enable us to draw casual association between parameters of OSA and PAC, whereas current observations are consistent with previous ones in resistant hypertension. Second, the number of women participants in clinical data was relatively smaller, which may explain, at least in part, the null association between PSG parameters and PAC. Third, in community-based data, OSA was diagnosed by questionnaires, which may have brought some bias to the results when considering the subjectivity of questionnaire-based diagnosis of a disease. Although these questionnaires can diagnose sleep disordered breathing, an umbrella term with a prevalence of 24.0%–83.8% (48–51), OSA is the most frequent type (52). In addition, No-SAS score has shown high sensitivity and specificity for OSA screening, compared with polysomnography, and has been validated in a Chinese population (31, 53). Moreover, it is not feasible to perform PSG in population-based studies due to its cost and technique dependence.

In conclusion, objective and subjective parameters of OSA showed significant independent linear association with PAC in clinical and general hypertensive and normotensive population, indicating that OSA is involved in aldosterone over-secretion and that aldosterone blockade might be a complementary therapy in patients with OSA and hypertension.

## Data availability statement

The data analyzed in this study is subject to the following licenses/restrictions: The data that support the findings of this study are available upon request from the corresponding author. Requests to access these datasets should be directed to lnanfang2016@sina.com; morale118@126.com/1017663289@qq.com.

## Ethics statement

The studies involving human participants were reviewed and approved by Ethics Committee at People’s Hospital of Xinjiang Uygur Autonomous Region. The patients/participants provided their written informed consent to participate in this study.

## Author contributions

NL and MH conceived the idea, designed the study, performed data collection and analysis and performed supervision of the study, participated in the paper writing, and gave crucial suggestions. HW and MH performed data collection and analysis and wrote the first draft. LG, MYL, WY, ML, LY, MML, AM, SL, ZW, ZX, LT, YL, QL and JH performed data collection and analysis and participated in sample measurement and in paper writing. All authors read and approved the final article. All authors contributed to the article and approved the submitted version.
