# Supplementary material for: Association of objective and subjective parameters of obstructive sleep apnea with plasma aldosterone concentration in 2,066 hypertensive and 25,368 general population
Source: Front Endocrinol (Lausanne). 2023 Jan 16;13:1016804. doi: 10.3389/fendo.2022.1016804 (PMC9884816; doi:10.3389/fendo.2022.1016804)
Supplement: Supplementary file 1 [file Table_1.docx]

| Sup Table 1. Comparison of log plasma aldosterone concentration by tertile of polysomnography sleep parameters in women participants with hypertension under no interfering agents with bonferroni correction for between group comparison. | | | | | | | | |
| --- | --- | --- | --- | --- | --- | --- | --- | --- |
|  | T1 | T2 | T3 | P | P1 | P2 | P3 | P trend |
| Log PAC |  |  |  |  |  |  |  |  |
| AHI tertile (n) | 1.12±0.24 | 1.13±0.23 | 1.13±0.23 | 0.682 | / | / | / | 0.491 |
| AI tertile (n) | 1.13±0.24 | 1.13±0.24 | 1.12±0.23 | 0.864 | / | / | / | 0.658 |
| HI tertile (n) | 1.12±0.24 | 1.13±0.23 | 1.13±0.23 | 0.877 | / | / | / | 0.755 |
| LSaO2 tertile (n) | 1.12±0.24 | 1.12±0.23 | 1.14±0.24 | 0.708 | / | / | / | 0.421 |
| MSaO2 tertile (n) | 1.12±0.24 | 1.12±0.24 | 1.12±0.24 | 0.870 | / | / | / | 0.786 |
| T90 tertile (n) | 1.14±0.23 | 1.13±0.25 | 1.12±0.23 | 0.694 | / | / | / | 0.394 |
| ODI3 tertile (n) | 1.12±0.24 | 1.14±0.24 | 1.12±0.24 | 0.980 | / | / | / | 0.678 |
| ODI4 tertile (n) | 1.11±0.24 | 1.14±0.24 | 1.13±0.23 | 0.741 | / | / | / | 0.482 |
| Max dur of hypoventilation tertile | 1.15±0.25 | 1.13±0.23 | 1.11±0.24 | 0.328 | / | / | / | 0.135 |
| Aver dur hypoventilation tertile | 1.15±0.24 | 1.12±0.24 | 1.12±0.24 | 0.229 | / | / | / | 0.130 |
| WASO | 1.14±0.22 | 1.13±0.25 | 1.12±0.24 | 0.493 | / | / | / | 0.235 |
| Arousal index | 1.14±0.22 | 1.12±0.24 | 1.13±0.25 | 0.533 | / | / | / | 0.429 |
| PAC:plasma aldosterone concentration; AHI:apnea hypopnea index; AI:apnea index; HI:hyponea index; LSaO2:lowest desaturation of oxygen; MSaO2:Mean oxygen saturation index; T90:time spent with oxygen saturation <90%; ODI3:oxygen desaturation index ≥3%; ODI4:oxygen desaturation index ≥4%; hypo: hypoventilation; WASO: wake after sleep onset. | | | | | | | | |

| Sup Table 2. Sample size by tertile of polysomnography sleep parameters in men and women participants with hypertension under no interfering agents. | | | | |
| --- | --- | --- | --- | --- |
| Tertiles of PSG parameters | overall | T1 | T2 | T3 |
| Men |  |  |  |  |
| Apnea hypopnea index tertile (n) | 1412 | <7.1 (410) | 7.1-20.6 (449) | ≥20.6 (553) |
| Apnea index tertile (n) | 1408 | <0.3 (356) | 0.3-3.9 (472) | ≥3.9 (580) |
| Hyponea index tertile (n) | 1408 | <5.3 (436) | 5.3-14.1 (466) | ≥14.1 (506) |
| Lowest desaturation of oxygen (n) | 1406 | ≥85 (505) | 79-85 (440) | <79 (461) |
| Mean oxygen saturation index tertile (n) | 1411 | ≥94 (515) | 92-94 (511) | <92 (385) |
| Time Spent with Oxygen Saturation <90% tertile (n) | 1403 | <3.54 (480) | 3.54-33.6 (466) | ≥33.6 (457) |
| Oxygen Desaturation Index ≥3% | 1403 | <22.5 (394) | 22.5-45.3 (464) | ≥45.3 (545) |
| Oxygen Desaturation Index ≥4% | 1403 | <11.2 (408) | 11.2-29.6 (451) | ≥29.6 (544) |
| Maximum duration of hypoventilation tertile (n) | 1403 | <28.6 (399) | 28.6-40.2 (465) | ≥40.2 (539) |
| Average duration of hypoventilation tertile (n) | 1403 | <18.2 (394) | 18.2-21.2 (474) | ≥21.2 (535) |
| Wake after sleep onset | 1403 | <40.0 (430) | 40.0-84.3 (488) | ≥84.3 (485) |
| Arousal index | 1403 | <10.0 (377) | 10.0-18.0 (519) | ≥18.0 (507) |
| Women |  |  |  |  |
| Apnea hypopnea index tertile (n) | 654 | <4.1 (216) | 4.1-13.1 (218) | ≥13.1 (220) |
| Apnea index tertile (n) | 651 | <0.1 (255) | 0.1-0.8 (170) | ≥0.8 (226) |
| Hyponea index tertile (n) | 651 | <3.5 (216) | 3.5-11.1 (216) | ≥0.8 (219) |
| Lowest desaturation of oxygen (n) | 654 | ≥86 (234) | 80-86 (231) | <80 (189) |
| Mean oxygen saturation index tertile (n) | 653 | ≥94 (275) | 92-94 (203) | <92 (175) |
| Time Spent with Oxygen Saturation <90% tertile (n) | 650 | <0.9 (195) | 0.9-18.0 (232) | ≥18.0 (223) |
| Oxygen Desaturation Index ≥3% | 650 | <14.2 (290) | 14.2-31.0 (222) | ≥31.0 (138) |
| Oxygen Desaturation Index ≥4% | 650 | <6.3 (276) | 6.3-17.5 (234) | ≥17.5 (140) |
| Maximum duration of hypoventilation tertile (n) | 650 | <24.4 (198) | 24.4-34.6 (226) | ≥34.6 (226) |
| Average duration of hypoventilation tertile (n) | 650 | <17.3 (194) | 17.3-20.0 (227) | ≥20.0 (229) |
| Wake after sleep onset | 650 | <43.5 (197) | 43.5-80.0 (226) | ≥80.0 (227) |
| Arousal index | 650 | <9.0 (155) | 9.0-16.0 (261) | ≥16.0 (234) |
| PAC:plasma aldosterone concentration. | | | | |

| Sup Table 3. Univariate liner regression for the relationship of Log-transformed parameters with Log PAC in men participants with no interfering agents. | | | |
| --- | --- | --- | --- |
|  | B, 95%CI, P | tolerance | VIF |
| Log Age | -0.129 (-0.249,-0.008), 0.036 | 0.84 | 1.18 |
| Log body mass index | 0.205 (-0.107,0.416), 0.058 | 0.58 | 1.71 |
| Cigarette consumption | -0.008 (-0.032,0.016), 0.513 | / | / |
| Alcohol intake | -0.009 (-0.032,0.015), 0.472 | / | / |
| Log Abdominal circumference (cm) | 0.293 (-0.129,0.428), 0.149 | / | / |
| Log ALT | 0.046 (-0.003,0.095), 0.065 | 0.95 | 1.05 |
| Log TC | 0.049 (-0.069,0.167), 0.416 | / | / |
| Log TG | 0.001 (-0.045,0.047), 0.955 | / | / |
| Log eGFR (ml/min/1.73m2) | -0.113 (-0.260,0.034), 0.132 | / | / |
| Log PRA | 0.121 (0.098,0.144), <0.001 | 0.90 | 1.11 |
| Log Fasting blood glucose (mmol/L) | 0.023 (-0.117,0.163), 0.748 | / | / |
| Log Serum potassium (mmol/L) | -0.697 (-1.026,-0.368), <0.001 | 0.96 | 1.05 |
| Log Serum sodium (mmol/L) | -0.049 (-1.542,1.444), 0.949 | / | / |
| Log Systolic blood pressure (mmHg) | 0.199 (-0.005,0.402), 0.055 | 0.53 | 1.90 |
| Log Diastolic blood pressure (mmHg) | 0.248 (0.061,0.435), 0.011 | 0.51 | 1.96 |
| Log 24 hour urinary potassium (mmol/l) | 0.220 (0.089,0.352), 0.001 | 0.94 | 1.07 |
| Log 24 hour urinary soudium (mmol/l) | -0.050 (-0.075,0.175), 0.431 | / | / |
| ALT: alanine aminotransferase; TC: total cholesterol; TG: triglyceride; eGFR: estimated glomerular filtration rate; PRA:plasma renin activity. | | | |

| Sup Table 4. Associated factors of PAC in univariate logistic regression and colinearity diagnosis in community-based sample. | | | |
| --- | --- | --- | --- |
| Variables | B, 95%CI, P | Tolerance | VIF |
| Age (years) | 1.08 (1.07,1.08), <0.001 | 0.653 | 1.532 |
| Systolic blood pressure (mmHg) | 1.04 (1.03,1.04), <0.001 | 0.396 | 2.524 |
| Diastolic blood pressure (mmHg) | 1.05 (1.047,1.052), 0.007 | 0.448 | 2.232 |
| Body mass index (kg/m^2^) | 1.36 (1.35,1.38), <0.001 | 0.328 | 3.046 |
| Abdominal circumference (cm) | 1.15 (1.146,1.154), <0.001 | 0.204 | 4.913 |
| Fasting blood glucose (mmol/L) | 1.17 (1.15,1.19), <0.001 | 0.935 | 1.070 |
| Serum creatinine | 1.01 (1.006,1.009), <0.001 | 0.978 | 1.023 |
| Total cholesterol (mmol/L) | 1.15 (1.13,1.18), <0.001 | 0.916 | 1.092 |
| Triglecyride (mmol/L) | 1.14 (1.11,1.16), <0.001 | 0.993 | 1.007 |
| Alanine aminotransferase (U/L) | 1.02 (1.01,1.02), <0.001 | 0.406 | 2.463 |
| Aspartate aminotransferase (U/L) | 1.01 (1.006,1.012), <0.001 | 0.417 | 2.397 |
| Education (≥9 vs <9 years) | 1.13 (1.07,1.20), <0.001 | 0.908 | 1.101 |
| Cigarette consumption (yes vs no) | 2.21 (2.21,2.34), <0.001 | 0.742 | 1.347 |
| Alcohol intake (yes vs no) | 2.54 (2.41,2.69), <0.001 | 0.744 | 1.345 |
| Cardiovascular disease (yes vs no) | 3.38 (3.02,3.79), <0.001 | 0.946 | 1.057 |
